# Comparative analysis of five protein-protein interaction corpora

**DOI:** 10.1186/1471-2105-9-S3-S6

**Published:** 2008-04-11

**Authors:** Sampo Pyysalo, Antti Airola, Juho Heimonen, Jari Björne, Filip Ginter, Tapio Salakoski

**Affiliations:** 1 Turku Centre for Computer Science (TUCS) and the Department of IT, University of Turku, Joukahaisenkatu 3-5, 20520 Turku, Finland

## Abstract

**Background:**

Growing interest in the application of natural language processing methods to biomedical text has led to an increasing number of corpora and methods targeting protein-protein interaction (PPI) extraction. However, there is no general consensus regarding PPI annotation and consequently resources are largely incompatible and methods are difficult to evaluate.

**Results:**

We present the first comparative evaluation of the diverse PPI corpora, performing quantitative evaluation using two separate information extraction methods as well as detailed statistical and qualitative analyses of their properties. For the evaluation, we unify the corpus PPI annotations to a shared level of information, consisting of undirected, untyped binary interactions of non-static types with no identification of the words specifying the interaction, no negations, and no interaction certainty.

We find that the F-score performance of a state-of-the-art PPI extraction method varies on average 19 percentage units and in some cases over 30 percentage units between the different evaluated corpora. The differences stemming from the choice of corpus can thus be substantially larger than differences between the performance of PPI extraction methods, which suggests definite limits on the ability to compare methods evaluated on different resources. We analyse a number of potential sources for these differences and identify factors explaining approximately half of the variance. We further suggest ways in which the difficulty of the PPI extraction tasks codified by different corpora can be determined to advance comparability. Our analysis also identifies points of agreement and disagreement in PPI corpus annotation that are rarely explicitly stated by the authors of the corpora.

**Conclusions:**

Our comparative analysis uncovers key similarities and differences between the diverse PPI corpora, thus taking an important step towards standardization. In the course of this study we have created a major practical contribution in converting the corpora into a shared format. The conversion software is freely available at .

## Background

Natural language processing (NLP) methods have recently been widely applied to improve access to the enormous and rapidly growing amount of information that is available mainly in the biomedical research literature. Protein-protein interactions (PPI) are the most widely studied information extraction (IE) target in the BioNLP field, with the key subproblem of protein name recognition being the most commonly addressed task.

Recent shared tasks and studies of biomedical named entities have increasingly clarified the concept of a protein name, brought about a rough consensus on how to annotate them, and established both the best-performing entity name recognition methods and their performance (see e.g. [1,2]). By contrast, little such standardization has occurred for PPI. Proposed PPI extraction methods are evaluated on test sets of widely differing sizes, domains and annotation schemes, and the reported results vary substantially, with, for example, recently proposed full parsing-based methods reporting F-scores ranging at least from 34% to 80% [3,4]. Even a shallow survey of PPI corpora suffices to establish that their annotations differ widely, yet no comparative study has so far been performed to quantify these differences or identify their source. Consequently, the BioNLP community faces a situation where it is difficult, if not impossible, to reliably identify the best published methods and techniques due to a lack of information on the comparability of their evaluated performance.

Lacking explicit, widely accepted definitions of PPI and their annotation, the best way to approach these topics is through the corpora produced by the groups studying PPI extraction. By analyzing corpora it is possible to identify points of agreement and disagreement regarding the definition of a protein-protein interaction and how they should be annotated, thus taking a step toward reaching a consensus on this topic. A comparative evaluation of PPI extraction methods on the various corpora could also complement broad single-corpus evaluation efforts such as BioCreative [2] and help determine the extent to which results of evaluations performed on different corpora are comparable and improve the ability to evaluate and compare methods for PPI extraction. This analysis and evaluation is the aim of this study. We survey five biomedical corpora manually annotated for PPI to identify their key similarities and differences. We identify the “greatest common factor” level of PPI annotation that is capable of capturing the main annotations of all the corpora, and we introduce normalizing transformations from the various corpora to a common format, thus allowing them to be combined into a large, multi-domain PPI corpus. Using the unified annotations and a re-implementation of a recently proposed state-of-the-art PPI extraction method as a benchmark, we perform an evaluation of the relative difficulty of the PPI extraction tasks codified by the corpora, providing the first quantitative comparative PPI corpus analysis. We then proceed to study the properties of the corpora contributing toward these differences, analyzing the potential sources of divergence and their importance.

## Results and discussion

In the following, we describe and present analysis results of the five selected corpora: AIMed, BioInfer, HPRD50, IEPA, and LLL. See the Materials and Methods section for further details regarding the corpora, their selection, and the evaluation methodology.

### Characterization of the corpora

Table 1 summarizes many of the characteristics of the corpora, determined by studying the publications and documentation describing the corpora as well as the corpora themselves. We first briefly discuss these general properties and their relevance to the PPI extraction task before proceeding to more detailed analyses. Note that while many corpora contain annotation for a broader class of entities than just proteins and a set of relationships broader than interactions, we will use “PPI” to refer to all of these annotations due to the pervasiveness of this term in the literature.

**Table 1:** Corpora

		AIMed	BioInfer	HPRD50	IEPA	LLL
	*size*	1955	1100	145	486	77

Entity	*scope*	human P/G	P/G/R and related	human P/G	Chemicals	P/G
	*coverage*	all occurrences	all occurrences	NER system	list of 16 names	list of 116 names
	*types*	no	111 types (ontology)	no	no	P/G

PPI	*types*	no	68 types (ontology)	no	no	3 types
	*binding*	no	yes	no	yes	no
	*directed*	no	yes	no	yes	yes
	*complex*	no	yes	no	no	no
	*negative*	no	yes	no	no	no
	*certainty*	no	no	yes	no	no

Legend:

Size: Number of sentences in the corpus

Entity scope: Types of the named entities identified in the corpus: (P)rotein, (G)ene, (R)NA

Entity coverage: Coverage of in-scope entity occurrences in each sentence

Entity types: Explicit identification of the type of the annotated named entity occurrences

PPI types: Explicit indication of the type of the annotated interactions

PPI binding: Identification of the specific text spans that entail the annotated interactions

PPI directed: Specification of the directionality of the interaction (typically identification of agent vs. patient roles)

PPI complex: Annotation includes nested or *n*-ary (for *n* > 2) interactions

PPI negative: Annotation of negative interactions

PPI certainty: Annotation of the levels of certainty, or speculativeness, of interactions

All five corpora contain annotation identifying entities such as proteins and genes in the text, but only LLL and BioInfer contain information regarding the types of the entities, and for LLL, this is limited to distinguishing between genes and proteins. More importantly for use in PPI extraction system development, only the AIMed and BioInfer corpora aim to include exhaustive annotation of entities of the types relevant to the corpus, while other corpora have based entity annotation on lists of entity names or named entity recognizer output.

The differences in interaction annotation are even greater than those in entity annotation: in particular, only the BioInfer and IEPA corpora contain information identifying the words stating an interaction, all but HPRD50 specify the direction of interactions, BioInfer alone contains complex or negative interactions, and only HPRD50 annotates different interaction certainties. Finally, BioInfer is the only corpus to contain annotation for static entity relations such as protein family membership. These properties dictate the greatest common factor for the purpose of unifying the corpora and evaluating them with a shared methodology: undirected, untyped interactions of non-static types with no text binding of words specifying the interaction, no complex structure, no negations, and no interaction certainty. We created custom software for each corpus, filtering and transforming its data into such a unifying subset (see the Materials and Methods section for more details). All results and discussion below concern these transformed versions of the corpora. Unfortunate as it may be that other information is discarded when unifying the corpora, this level of annotation is also a reflection of the state of the art in biomedical IE methods, and doesn't presently limit the applicability of the resulting dataset.

### PPI extraction performance

We next present evaluation results for the unified corpora using two PPI extraction methods as benchmarks: a simple co-occurrence based method and RelEx [4], a full parsing-based IE system with state-of-the-art performance. Our use of these methods is not primarily intended as an evaluation of these methods, but rather of the corpora. We use the gold standard named entities as annotated in each corpus with both methods.

The performance of the two methods on the various corpora is given in Table 2. Note that for simple co-occurrence, all pairwise interactions are extracted, but all self-interactions are missed — thus recall is trivially 100% for corpora that have no self-interactions and lower for those that do. Even though notable differences in F-score results were expected, their magnitude is striking: for example, on the LLL corpus F-score error is just 34%, while on AIMed and BioInfer it is over 70%.

**Table 2:** PPI extraction performance

Corpus	Co-occurrence			RelEx		
	P	R	F	P	R	F
AIMed	0.17	0.95	0.29	0.40	0.50	0.44
BioInfer	0.13	0.99	0.23	0.39	0.45	0.41
HPRD50	0.38	1.0	0.55	0.76	0.64	0.69
IEPA	0.41	1.0	0.58	0.74	0.61	0.67
LLL	0.50	1.0	0.66	0.82	0.72	0.77

(P)recision, (R)ecall, and (F)-score for the co-occurrence and RelEx methods on the various corpora.

**Figure 1:**
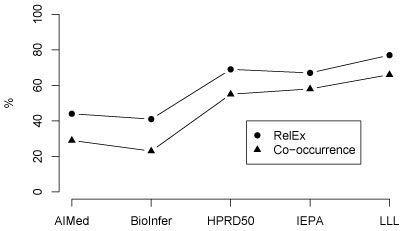
RelEx and co-occurrence F-scores for the five corpora

For RelEx, we find that F-score performance is better for all corpora than for co-occurrence. We note that the performance of the two methods correlates closely (see Figure 1); the relative reduction in F-score error from the co-occurrence method to RelEx is relatively consistent, ranging from 21–32% between the different corpora. This highlights the value of applying a baseline method to establish the relative difficulty of an extraction task. However, great differences remain in performance between corpora: for example, the use of the advanced method does not reduce the over twofold increase in error from LLL to the AIMed and BioInfer corpora. Given these results, it is clear that differences stemming from the choice of corpus can be substantially larger than differences in the performance of PPI extraction methods. Indeed, the average absolute F-score difference between the co-occurrence and RelEx methods is just 13%, while the average difference between pairs of these corpora as measured by RelEx is a remarkably large 19%. Thus, here *the choice of corpus has a larger effect on the result than the choice between a naive PPI extraction method and an advanced one*. This has the immediate and important implication that results for methods evaluated on different corpora should in general not be directly compared, and suggests limits on how meaningful such comparisons are when the comparability of the corpora has not been established. This result can also be seen as calling into question whether F-score is a meaningful performance measure for PPI extraction, but we expect that its position as a *de facto* standard is unlikely to be shaken.

### Corpus statistics

In the following, we aim to identify some of the factors behind the broadly diverging PPI extraction performance between corpora. Table 3 shows a number of key statistics measured from the corpora. We first note that while all corpora except AIMed have at least one annotated entity for each sentence, the fraction of sentences with no annotated interactions varies from zero to more than two thirds. This may reflect the corpus authors' different views regarding the appropriate starting point for extraction, in particular on how aggressively non-relevant sentences can be filtered out. While the fraction of sentences without interactions appears to be loosely connected to RelEx performance, the correlation is not statistically significant.

**Table 3:** Corpus statistics

Corpus	Per sentence average number of				Fraction of sentences with	
	Tokens	Entities	Entity pairs	Interactions	No entities	No interactions
AIMed	25.2	2.2	3.0	0.5	18%	69%
BioInfer	31.3	4.2	9.4	1.3	~0%	48%
HPRD50	26.1	2.8	3.0	1.1	0%	38%
IEPA	32.2	2.3	1.7	0.7	0%	37%
LLL	29.6	3.1	4.3	2.1	0%	0%

More significantly, we observe that the average number of annotated entities per sentence varies almost twofold from just above two to more than four, translating to even greater differences in the average number of entity pairs: here we find e.g. an over fivefold difference between IEPA and BioInfer. The average number of annotated interactions per sentence also varies considerably, from just half on AIMed to more than two on LLL. These last two statistics are connected to co-occurrence based PPI extraction performance through a simple relationship: the average number of interactions divided by the average number of entity pairs per sentence (below abbreviated *I/EP*) equals the precision of the co-occurrence method evaluated above. Further, as recall for this method can be approximated to be 1 (ignoring rare self-interactions), $F\;=\;\frac{2pr}{p+r}\;\approx \;2\text{ × I/EP / }\left(\text{1 + I/EP}\right)\text{,}$ where *p* is precision and *r* recall. *I/EP* correlates closely with RelEx performance, although due in part to the conservativeness of the significance test we apply, this correlation is only weakly significant (*p* ≈ 0.08). We propose that *I/EP* may serve as a rough, easily computable baseline for determining the comparative difficulty of PPI extraction tasks across corpora. As more proteins are annotated, we would not expect *I* to grow more than linearly, while *EP* grows quadratically.

**Figure 2:**
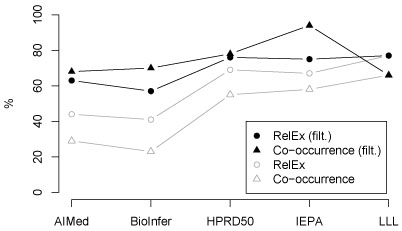
**RelEx and co-occurrence F-scores for the filtered version of the corpora.** Unfiltered results given in Figure 1 shown in gray for reference.

These results suggest that much of the difference between the corpora is due to the simple factors *I* and *EP*, but do not show it conclusively—it is possible that the correlation with RelEx performance is due to other factors or only coincidental. We can test the effect on RelEx performance by removing from the annotation proteins for which there is no interaction, thus reducing *EP*. The result of performing this filtering on the corpora is shown in Table 4 and illustrated in Figure 2. The filtering results in a substantial narrowing of RelEx performance differences between the corpora, bringing the average difference down from 19% to 11%. Thus, we find that *annotation of proteins for which there is no annotated interaction determines almost half of the performance difference between corpora*. Remarkably, filtering also causes the simple co-occurrence method to outperform the more advanced method on all corpora except LLL, where no entities are filtered. This further indicates that evaluation on corpora that mainly include annotation for interacting entities leads to a substantial bias toward favoring methods that emphasize recall over precision. While the above analysis explains almost half of the average performance difference between corpora, an over 10% average pairwise difference remains. Further, a pure statistical analysis cannot shed light onto the key question of where the differences measured by the statistics arise from. To illuminate these issues, we next proceed to a qualitative analysis of the corpora.

**Table 4:** PPI extraction performance on filtered corpora

Corpus	Co-occurrence				RelEx			
	P	ΔP	F	ΔF	P	ΔP	F	ΔF
AIMed	0.53	0.36	0.68	0.39	0.85	0.45	0.63	0.19
BioInfer	0.53	0.40	0.70	0.47	0.78	0.39	0.57	0.16
HPRD50	0.64	0.26	0.78	0.23	0.93	0.17	0.76	0.07
IEPA	0.88	0.47	0.94	0.36	1.00	0.26	0.75	0.08
LLL	0.50	0.00	0.66	0.00	0.82	0.00	0.77	0.00

Precision and F-score for the co-occurrence and RelEx methods on the corpora with only entities that participate in an interaction preserved. Recall is not shown as it is unaffected by this modification. The Δ columns show absolute difference to results without filtering.

### Qualitative analysis

To examine how each of the five chosen corpora defines a protein-protein interaction and to what extent the annotations agree, a random sample of 50 interactions from each corpus was examined by two annotators with expertise in biology and corpus annotation. Each interaction was analysed with respect to five aspects: type, directness, explicitness, certainty, and polarity (defined in detail in the Materials and Methods section).

**Figure 3:**
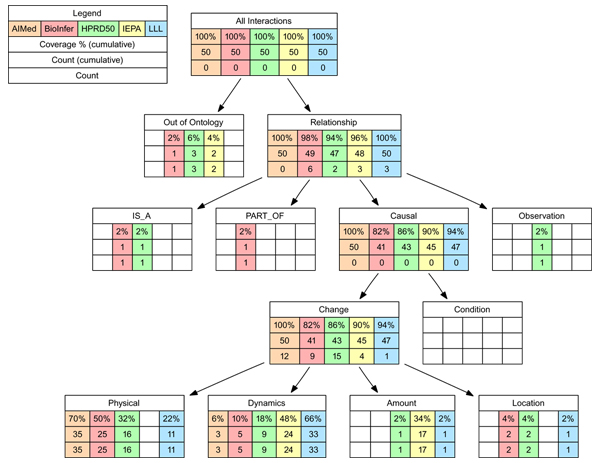
**Distribution of interaction types in the corpora as mapped to the BioInfer ontology.** In cases where the reason why an interaction was annotated could not be identified the supplemental *Out of Ontology* type was assigned. Empty cells represent zero count.

Figure 3 shows the distribution of interaction types in the various corpora across the BioInfer ontology — for definitions of the types, see [5]. There are many similarities and some important differences in the distributions. We note that a clear majority of all interactions fall under the *Causal-Change* subtree of the BioInfer ontology, with the BioInfer corpus having the most exceptions to this rule. The interactions in the *Causal-Change* subtree correspond to events occurring as part of biochemical processes in living cells as opposed to static, non-event relations such as family membership and structural similarity. This supports our understanding that the corpora do not contain notable annotation of static relations between entities, with the exception of BioInfer, from which most static relations were filtered. The most common specific type were interactions related to a *Change* in the *Physical* properties of entities, with the IEPA corpus a notable outlier in containing no interactions of this type. We observe that the corpus type distributions, while surprisingly different, appear to mostly follow the definitions of “interaction” in the corpora, with e.g. IEPA interactions focusing on *Change* in *Amount* and *Dynamics* (see the Corpora section).

To identify similarities in the type distributions of the corpora, we evaluated the correlation of the interaction type frequencies, using the non-cumulative counts (see Figure 3). This analysis suggests that the AIMed, BioInfer and HPRD50 corpora have the most similar type distributions, with IEPA and, to a lesser extent, LLL standing out with different distributions. However, this clustering cuts across any trends that could be seen in the PPI extraction performance results, with HPRD50 in particular clustering with IEPA and LLL on performance but with AIMed and BioInfer with respect to types. This analysis does thus not support the hypothesis that interaction type alone would be a deciding factor for PPI extraction performance.

We now turn to the results of the analysis regarding the directness, explicitness, certainty, and polarity of the corpus interactions (see Table 5). The clearest universal trend is that almost all annotated interactions in all analysed corpora are positive; as noted above, explicitly marked negative statements in BioInfer were filtered out prior to analysis. Machine-learning based PPI extraction systems trained and tested on such data will aim not to extract negative interactions, and several rule-based systems explicitly avoid their extraction (see e.g. [4,6]). Thus, omitting negative statements is currently appropriate in evaluation—systems should be evaluated with respect to their intended coverage. We also observe that a great majority of all annotated interactions in all corpora were definite. However, in the light of the estimate of Light et al. [7] that only 11% of sentences in PubMed abstracts contain speculation this is more likely due to the relative rarity of speculative statements than to a decision to avoid annotating them. Indeed, the results suggest that all corpora annotate speculative statements roughly as frequently as they would be expected to appear.

**Table 5:** Qualitative analysis results

Corpus	Explicit	Direct	Definite	Positive
AIMed	52%	72%	92%	98%
BioInfer	67%	45%	93%	100%
HPRD50	75%	53%	81%	92%
IEPA	86%	6%	92%	100%
LLL	73%	24%	94%	100%

Results of the analysis of 50 interactions per corpus with respect to their directness, explicitness, certainty, and polarity. Results are given as fraction of analysed interactions that were not out-of-ontology (see Figure 3) and were identified as having a given property by two annotators. Cases where annotators disagreed were counted as half a point.

By contrast to these aspects, the results for directness and explicitness are less uniform. The greatest divergence is in directness, where only very few interactions in IEPA are direct, a clear minority in LLL, about half in BioInfer and HPRD50, and a clear majority in AIMed. This reflects in part the types of the annotated interactions, but it also makes it clear that unlike some efforts to extract interaction networks (e.g. [8]), the corpora do not aim to separate direct interactions from indirect ones. The fraction of explicit statements ranges from about half on AIMed to almost 90% on IEPA, and here one might tentatively hope to recognize a connection to PPI extraction performance, with AIMed and BioInfer having low scores and the three other corpora high scores on both metrics. However, this correlation was not statistically significant, possibly in part due to small sample size and the presence of confounding factors.

In conclusion, the qualitative analyses uncovered several similarities and differences between the corpora with respect to many of the analysed characteristics, identifying some of the often unstated rules applied in PPI annotation. Nevertheless, although we studied several potentially relevant factors, the analysis did not identify a clear reason for the remaining over 10% average PPI extraction performance difference between corpora. These results suggest that the remaining differences may, in fact, be largely due to idiosyncrasies of the corpora instead of general properties. As an example of such a feature, we note that approximately 5% of interactions in the AIMed corpus are self-interactions (a single protein interacting with itself), which the methods applied here do not attempt to extract — and essentially no self-interactions occur in the other corpora. Further regularities might also be uncovered by the use of additional benchmark methods, and we expect analysis applying machine-learning approaches to be a particularly fruitful avenue of further research in comparative PPI corpus analysis.

### Recommendations and best practices

The results of our evaluation underline the importance of establishing the relative difficulty of the extraction tasks represented by corpora. Based on these results, we strongly recommend that corpus creators and PPI extraction method evaluations measure this difficulty by reporting performance for an established baseline method or, minimally, simple corpus statistics such as* I/EP*. The use of standard datasets, metrics, and evaluation protocols is naturally also important. We further recommend evaluation on several corpus resources: with the five corpora considered in this study now available in a common format, we believe that future PPI extraction system evaluations would be enhanced by reporting performance for all of these corpora, thus obtaining broader insight into system performance.

In corpus annotation, we would stress in particular the value of annotation manuals or similar documentation and the use of standoff annotation and XML formats in making corpora accessible to users. Cohen et al. [9] discuss in detail these and other valuable recommendations for corpus development based on an analysis of several biomedical domain corpora. As one of the main outcomes of this study, we established a clear relationship between the annotation of non-interacting entities and the performance of the PPI system: performance on corpora that annotate all entities of the relevant type, arguably a realistic model of the real PPI extraction task, was considerably lower. Based on this finding, we recommend the annotation of all relevant entities in subsequent corpus development projects. Finally, in moving forward from the current, relatively low level of shared corpus annotation, we would like to emphasize the value of annotating the direction of interactions as well as specific annotation for negative statements of interactions. These practices would benefit both evaluation and users of the extracted interactions.

## Conclusions

We have presented the first comparative evaluation of PPI corpora, studying the AIMed, BioInfer, HPRD50, IEPA, and LLL corpora. Our evaluation showed great divergence in PPI extraction performance between corpora, finding an average F-score difference of 19% for a recently proposed extraction method. We studied the properties of the corpora to identify factors causing these differences, and established that approximately half of the performance difference between corpora stems from the annotation given to non-interacting entities. We also performed a detailed qualitative analysis of the corpora, uncovering notable differences in the distribution of interaction types and identifying often-unstated points of agreement and disagreement in annotation regarding negation, speculative statements, the explicitness of annotated statements, and the directness of the corpus interactions.

As a major practical contribution of this study, we have defined a conversion for each corpus into a shared annotation scheme, allowing the diverse PPI corpora to be merged into a large, multi-domain corpus. The conversion programs are freely available at .

## Materials and methods

### Corpora

For the survey, we selected freely available PPI corpora with specifically identified named entities and manually annotated interactions. We further required that negative examples of PPI be either explicitly marked or that they can be validly generated under the closed-world assumption. These criteria are approximately minimal for evaluation using the general type of extraction methods applied in this study. We selected all corpora fulfilling these requirements that we were aware of, including those that were included at the time of selection in the public resource collecting information regarding BioNLP corpora maintained by Cohen et al. [9,10]. The following corpora were selected: the large recently introduced PPI evaluation corpora AIMed [11] and BioInfer [5], the HPRD50 corpus annotated by Fundel et al. [4], the *IEPA* corpus [12], and the PPI corpus produced for the LLL challenge [13]. In the following, we briefly describe each of the corpora.

*AIMed* is a corpus created for PPI extraction method comparison [11,14]. The corpus was created from 200 PubMed abstracts identified by the Database of Interacting Proteins (DIP) [15] as containing protein-protein interactions. The abstracts were subsequently manually annotated for interactions between human genes and proteins. In addition to the 200 PPI abstracts, further 30 abstracts containing no PPIs were added to the corpus as negative examples. The current release of corpus consists of 225 of these abstracts.

*BioInfer* was created as a corpus for training and testing PPI extraction programs [5,16]. The corpus consists of sentences from PubMed abstracts that contain at least one pair of interacting proteins as defined by the Database of Interacting Proteins. A random subset of these sentences was annotated by the corpus authors for all entities of the protein, gene and RNA types, as well as related entity types such as processes and properties when these were involved in annotated relationships. All of the interactions between these entities were annotated, including static relations.

The *HPRD50* corpus was created as a test set for the RelEx system [4,17]. It was created from sentences from 50 abstracts referenced by the Human Protein Reference Database (HPRD). Human gene and protein names were automatically identified in these sentences with the ProMiner software. Direct physical interactions, regulatory relations and physical modifications between these entities were then annotated.

The *IEPA* corpus [12,18] was constructed from sentences from PubMed abstracts, each containing a specific pair of co-occurring chemicals. The ten pairs of chemicals—the majority of which were proteins—were chosen to represent diverse biological research topics. In each sentence, interactions between the given two entities were manually annotated, with an interaction being defined as “*a direct or indirect influence of one on the quantity or activity of the other*” [12].

The *LLL* corpus was created as the shared dataset for the Learning Language in Logic 2005 (LLL05) challenge [13,19]. The domain of LLL is gene interactions of Bacillus subtilis. The interactions are defined as agent/target pairs, where agent is a protein and target is a gene. The corpus contains three types of interaction: explicit action, binding of a protein on the promoter of the target gene, and membership in a regulon family.

### Corpus conversion

The unified format to which the corpora were transformed follows the standoff annotation principle, where the original sentence text is preserved and the entities are identified through character offsets. The corpora in the unified format are stored in XML files with a very simple structure.

The transformation of the corpora into the unified format is a tedious process requiring significant efforts. This is due to the often highly idiosyncratic native formats of the corpora requiring the development of relatively complex transformation programs and, in several cases, manual intervention to resolve ambiguity present in the native format. This work was in many cases very similar to the refactoring efforts described in detail in [20].

The conversion for the BioInfer corpus was particularly challenging due to its more complex interactions and broader scope than either the other corpora or the extraction methods. To convert BioInfer, we removed interactions annotated as negative, flattened nested entities, transformed complex interactions into binary according to a set of custom-developed rules so that only pairs of entities take part in interactions, and finally narrowed the set of interactions to the *Causal-Change* subtree of the BioInfer relationship type ontology.

We provide the transformation programs for all of the corpora under an open-source license, enabling other researchers to access the five PPI corpora in this study in a unified format [21].

### Evaluation methods and metrics

We use the precision, recall and F-score metrics, which are nearly universally applied in PPI extraction system evaluation. However, these metrics can be measured in different ways, potentially leading to considerably different results. We measured extraction accuracy according to the following rules:

*No directionality*: REL(*P*_1_,*P*_2_) is considered equivalent to REL(*P*_2_,*P*_1_).

*No interaction type*: it is not necessary to identify the type of an interaction.

*Entity identity*: REL(*P*_1_,*P*_2_) does not match REL(*P*_1_,*P*′_2_) unless *P*_2_ = *P*′_2_, even if *P*_2_ and *P*′_2_ are different occurrences of the same protein name.

We applied a simple co-occurrence method and a full parsing-based method, described in the following.

#### Co-occurrence method

One very simple PPI extraction method is to assign a relationship between all annotated entities co-occurring within a sentence. While some corpora include self-interactions (i.e. REL(*P*_1_,*P*_1_)), we do not extract such “co-occurrences” as this would have a detrimental effect on overall extraction performance. Despite their simplicity, co-occurrence methods have been successfully applied to many information extraction tasks in the biomedical domain (see e.g. [22]) and provide a relevant baseline for more advanced methods.

#### RelEx

**Figure 4:**
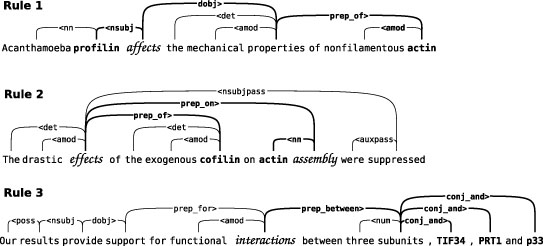
**RelEx interaction extraction rules**. The three RelEx rules search the dependency graph for paths between protein names indicating an interaction. Found paths are filtered according to criteria such as whether certain interaction terms appear in them. In the examples the protein names are marked in bold, and the possible interaction words in italics.  Rule 1 extracts **protein**_1_-*relation*-**protein**_2_ structures from the dependency graph. In the basic case the rule requires that the path includes a subject dependency (e.g. **nsubj**). Rule 2 extracts relations that are represented by a sequence of noun phrases connected to each other via prepositional dependencies (**prep**). Rule 3 searches for structures of the type *relation*-between-**protein**_1_-and-**protein**_2_ connected through **prep_between** and **conj_and** dependencies.

The RelEx method of Fundel et al. is a full parsing-based PPI extraction method with state-of-the-art performance [4]. Due to the generality of the system's concept of relation, it is not constrained to any specific subdomain of PPI-interactions. The fact that RelEx is based on simple principles and its performance has been tested on publicly available datasets makes reimplementing it a feasible task. RelEx is also purely rule-based and therefore does not require per-domain training data, which is beneficial for evaluating smaller corpora as the entire corpus can be used for evaluation and results are not skewed by differing amounts of training data. Three example interactions corresponding to the three RelEx rules are presented in Figure 4. For further details on the system we refer to [4].

We next give a brief overview of our implementation and application of RelEx. Similarly to the setup described by Fundel et al., we apply the MedPost tagger [23] and the Stanford Lexicalized Parser (Version 1.5.1) [24] to generate a dependency parse graph for each sentence. Instead of using an external noun-phrase chunker as in the original implementation, we base the chunking on an analysis of the dependency graph. The chunking information is merged to the dependency graphs, which are then subjected to parallel analysis according to the RelEx rules. The rules extract possible interaction paths consisting of words and dependencies that connect named entity pairs. In the post filtering phase, relations that are deemed to be negated as well as those that do not contain certain relation terms are filtered out. Lacking a published reference implementation of RelEx, the performance of our system diverges slightly from that of the original implementation by Fundel et al. We do not believe this to affect the suitability of our implementation for comparing the characteristics of different PPI corpora.

### Qualitative analysis

In this section we briefly define the methods and definitions used to perform the qualitative corpus analysis. We measured the distribution of the types of the annotated interactions by mapping them into the BioInfer relationship ontology. We used this ontology because of its broad coverage of protein-protein interaction types [5] and because its hierarchical structure allows aggregation of types at various levels of specificity. We assigned to each interaction the most specific applicable type. If no appropriate type was found, the interaction was marked out of the ontology as the type *Out of Ontology*.

*Directness* indicates whether the interaction involves physical contact of the proteins. This definition follows that in [8]. *Explicitness* reflects the way in which the interaction is stated. An explicitly stated interaction is one that can be understood without knowledge of biology-specific terms and inference supported by external biological knowledge. *Certainty* describes whether an interaction is stated in a definite manner or speculatively, with qualifiers such as *may*. We apply a binary distinction between speculative and definite statements, following the guidelines in [7]. *Polarity* refers to whether the statement asserts the existence or the non-existence of an interaction. We annotate negated statements following the annotation guidelines in [25]. While there are no widely accepted standards for annotating any of these aspects, we consider the guidelines referenced above as offering good candidates for standardization.

### Correlation estimate

Throughout the paper, correlation refers to Kendall's correlation coefficient and the associated test with null hypothesis being that the correlation coefficient equals zero. The *p* value is computed using the standard Best and Gipps algorithm as implemented in the R statistical package [26,27]. We use *p* = 0.05 as the cut-off value. We note that these statistics are very conservative due to the fact that they do not make any assumptions regarding the distribution of the results and do not consider their magnitude.

## Competing interests

The authors declare that they have no competing interests.

## Authors' contributions

FG, JB, JH and SP created the conversions of the corpora to the unified format. AA implemented the PPI extraction methods, and all authors participated in the analyses. SP was the main author of the manuscript with contributions from all other authors, all of whom read and approved the final version.
